# Cellular Processing of Myocilin

**DOI:** 10.1371/journal.pone.0092845

**Published:** 2014-04-14

**Authors:** Ye Qiu, Xiang Shen, Rajalekshmy Shyam, Beatrice Y. J. T. Yue, Hongyu Ying

**Affiliations:** Department of Ophthalmology and Visual Sciences, University of Illinois at Chicago College of Medicine, Chicago, Illinois, United States of America; University of Rochester, United States of America

## Abstract

**Background:**

Myocilin (*MYOC*) is a gene linked directly to juvenile- and adult-onset open angle glaucoma. Mutations including Pro370Leu (P370L) and Gln368stop (Q368X) have been identified in patients. In the present study, we investigated the processing of myocilin in human trabecular meshwork (TM) cells as well as in inducible, stable RGC5 cell lines.

**Methodology/Principal Findings:**

The turnover and photoactivation experiments revealed that the endogenous myocilin in human trabecular meshwork (TM) cells was a short-lived protein. It was found that the endogenous myocilin level in TM cells was increased by treatment of lysosomal and proteasomal inhibitors, but not by autophagic inhibitor. Multiple bands immunoreactive to anti-ubiquitin were seen in the myocilin pull down, indicating that myocilin was ubiquitinated. In inducible cell lines, the turnover rate of overexpressed wild-type and mutant P370L and Q368X myocilin-GFP fusion proteins was much prolonged. The proteasome function was compromised and autophagy was induced. A decreased PSMB5 level and an increased level of autophagic marker, LC3, were demonstrated.

**Conclusions/Significance:**

The current study provided evidence that in normal homeostatic situation, the turnover of endogenous myocilin involves ubiquitin-proteasome and lysosomal pathways. When myocilin was upregulated or mutated, the ubiquitin-proteasome function is compromised and autophagy is induced. Knowledge of the degradation pathways acting on myocilin can help in design of novel therapeutic strategies for myocilin-related glaucoma.

## Introduction

Glaucoma is one of the leading causes of irreversible blindness worldwide [1] characterized by progressive loss of retinal ganglion cells (RGCs) and their axons, as well as cupping of the optic nerve head. The most common form of this disease, primary open angle glaucoma (POAG), is frequently associated with elevated intraocular pressure (IOP) [2]. The IOP is controlled by a balance between the production and outflow of the aqueous humor contained in the anterior chamber of the eye. The trabecular meshwork (TM), a specialized tissue located next to the cornea, is the major site for regulation of the aqueous humor outflow [3]. Dysfunction of TM cells and accumulation of the extracellular matrix materials in TM tissues are believed to be factors contributing to IOP elevation and glaucoma development [3], [4].

POAG is highly heterogeneous, caused by several susceptibility genes [5], [6] and perhaps also environmental factors [7]. To date, candidate genes including myocilin (*MYOC*, also called *GLC1A*) [8], [9] and optineurin [10], [11] have been identified.

*MYOC* is the first gene identified for both juvenile- and adult-onset POAG [9]. It was originally cloned from cultured human TM cells after prolonged treatment of dexamethasone [12], [13]. The human *MYOC* gene encodes a 55-/57-kilodalton (kDa) acidic glycoprotein of 504 amino acids. Sequence analysis has revealed an amino (N)-terminal coiled coil domain (also known as nonmuscle myosin-like domain) containing therein a leucine zipper motif [14], a signal peptide sequence [2], a central linker region, and a carboxyl (C)-terminal olfactomedin-like domain. With a signal peptide sequence at the N-terminus, myocilin is, at least in part, secreted via the traditional secretory pathway [12], [13]. Myocilin has additionally been shown to associate with exosome-like vesicles and may also use this alternative mechanism to be released from the cell [14], [15].

Myocilin is a glycoprotein, N-glycosylated at amino acid residues 57–59 (Asn-Glu-Ser) [14]. When subjected to membrane protein extraction, myocilin in TM cell lysates distributed largely in the hydrophobic fraction in association with membranes [16]. The myocilin protein has in addition been reported to be cleaved into a 20 kDa N-terminal fragment and a 35 kDa C-terminal fragment both *in vitro* and *in vivo* [17], [18] The proteolytic processing of myocilin is suggested to have a role in regulating its molecular interactions [17], [19].

Myocilin interacts with itself at sites of the leucine zipper/coiled coil domain to form dimers and possibly multimers [14], [17], [20], [22]. It also interacts with a number of proteins including flotillin-1(a structural protein of lipid rafts), optimedin, extracellular proteins such as fibronectin and fibrillin-1, as well as matricellular proteins hevin and SPARC [14], [19], [21], [23].

Mutations of myocilin were found in 2–4% of POAG patients [6], [9]. More than 70 mutations in myocilin have been reported [6]. The disease-causing ones among them are located predominantly in the olfactomedin-like domain [6]. Pro370Leu (P370L), a missense mutation, is responsible for one of the most severe glaucoma phenotypes [24]–[26]. Gln368Stop (Q368X) is the most common myocilin mutation reported in POAG patients [27]. With nonsense mutation at amino acid residue 368, it generates a truncated protein of 367 amino acid length. Contrasting to the wild-type myocilin, mutants with mutations in the olfactomedin-like domain, however, are not secreted [28], [29]. They are retained in the cells, aggregating to induce endoplasmic reticulum (ER) stress and unfold protein response (UPR) [30].

Proteins are continually synthesized and degraded. Proteolysis is a crucially important regulatory mechanism that determines the protein turnover rates, maintains the protein quality control, and controls fundamental cellular processes including cell cycle, and signaling [31]. In eukaryotic cells, the machineries for protein processing encompass the autophagy-independent lysosomal [32], [33], proteasomal [31], [34], [35], and autophagy-dependent lysosomal or autophagosomal systems [36], [37].

In the present investigation, we examined the involvement of these pathways in processing of the endogenous myocilin in human TM cells. Photoactivation approach [38] was also employed to monitor the turnover of myocilin in TM cells. In addition, RGC5, a cell line recently verified to be mouse retinal photoreceptor 661 W [39], was used as a vehicle, not as a surrogate for retinal ganglion cells (RGCs), to facilitate studies of myocilin and its mutants. Tetracycline-inducible (Tet-on) RGC5 stable cell lines that express, upon doxycycline (Dox) induction, green fluorescence protein (GFP)-tagged wild-type and mutant (P370L and Q368X) myocilin were created [40]. The turnover of the overexpressed myocilin-GFP fusion proteins was further examined in these cells.

## Results

### Turnover of endogenous myocilin

**Figure 1:**
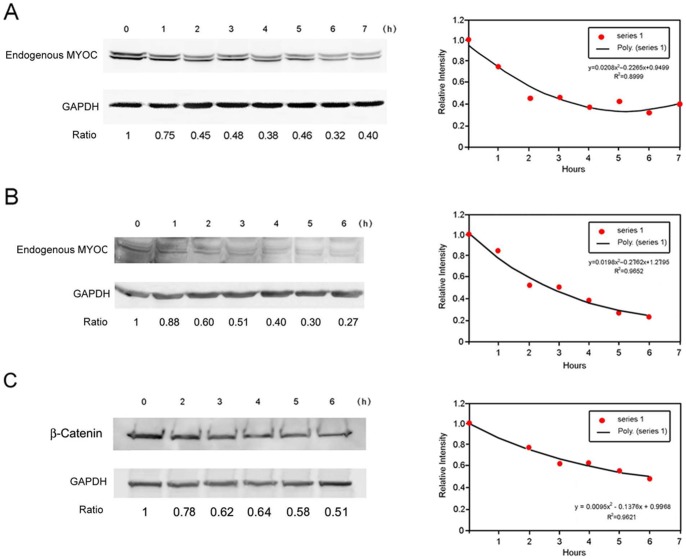
Turnover of the endogenous myocilin protein and β-catenin. **A**. Human TM cells from different donors were treated with CHX (5 µg/ml) and monesin (2 µM). Cells were harvested at various time points thereafter and the level of endogenous myocilin was determined by Western blotting. The GAPDH level was used for protein loading control. The level of the endogenous myocilin was dropped to about 50% 3 h after CHX and monesin treatment, indicating that myocilin is a short-lived protein. One representative experiment is presented. Ratios between levels of the endogenous myocilin and those of GAPDH are presented (left panel), which were plotted against time to estimate the myocilin half-life (right panel). **B**. The turnover of the endogenous myocilin in RGC5 cells. Experiments were done as described in **A**. The half-life was estimated to be about 3 h. **C**. The turnover of β-catenin, a known short-lived protein, in RGC5 cells. Its half-life was found to be approximately 6 h, comparable to that reported previously [41]. Experiments were repeated at least 3 times, yielding similar results.

The turnover experiments showed that the level of the endogenous myocilin, relative to that of glyceraldehyde 3-phosphate dehydrogenase (GAPDH), was dropped to about 50% 3 h after treatment of cycloheximide (CHX) to inhibit protein synthesis as well as monesin to block protein secretion in human TM cells. Myocilin is thus a short-lived protein with an estimated 3 h half-life (Fig. 1A). Similar to that in TM cells, the half-life of the endogenous myocilin in RGC5 cells was found to be around 3 h (Fig. 1B). Parallel control analysis of a known short-lived protein, β-catenin, in RGC5 cells showed a half-life of approximately 6 h (Fig. 1C), as was reported previously [41].

### Effects of inhibitors on myocilin levels in human TM cells

**Figure 2:**
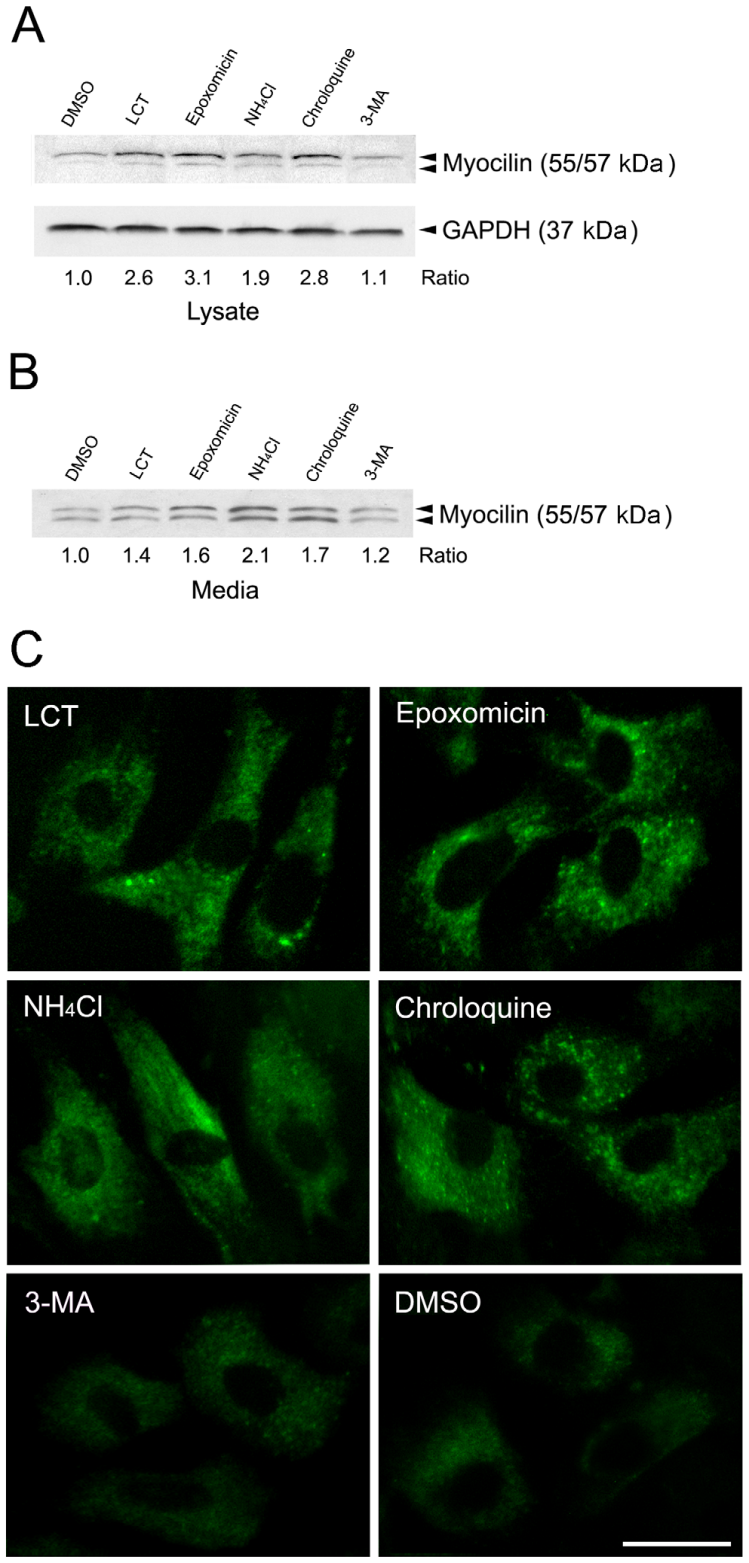
Effects of inhibitors on levels of endogenous myocilin. **A**. Western blotting. Human TM cells were treated for 16 h with vehicle DMSO or H_2_O (not shown), or proteasomal (LCT and epoxomicin), lysosomal (NH_4_Cl and chloroquin) or autophagic (3-MA) inhibitors. Proteins (25 µg) in cell lysates were immunoblotted with anti-myocilin or anti-GAPDH. Densitometry was performed. The myocilin/GAPDH relative to the DMSO control ratios are presented. **B**. Equal aliquots of media were immunoblotted with anti-myocilin. The level of myocilin in the media was normalized to that of the DMSO control. **C**. Immunofluorescence for myocilin. TM cells treated as above with the various inhibitors were fixed and immunostained for myocilin. All experiments were repeated at least 3 times, yielding similar results. Scale bar, 20 µm.

Human TM cells were treated for 16 h with vehicle dimethyl sulfoxide (DMSO) or H_2_O, or proteasomal (lactacystin or LCT and epoxomicin), lysosomal (NH_4_Cl or chloroquin), or autophagic (3-methyadenine or 3-MA) inhibitors. The cell lysates and media were collected. LCT is a proteasomal inhibitor but it also inhibits enzymes such as cathepsin A. Epoxomicin on the other hand is a potent and specific proteasomal inhibitor. NH_4_Cl is a lysosomotropic weak base that blocks the intralysosomal degradation of macromolecules via inhibition of the acidification of the endosome-lysosome system [42]. It does not affect enzyme activities. 3-MA inhibits class III phosphatidylinositol 3-kinase (PI3K) that is essential for autophagosome formation, as well as other classes of PI3K. It is used as an effective and selective drug to inhibit autophagy degradation. At 5 mM, it has no detectable effects on other proteolytic pathways [43]. Proteins in cell lysates were immunoblotted with monoclonal anti-myocilin [44] or polyclonal anti-GAPDH. Densitometry was performed and the myocilin/GAPDH relative to the vehicle control ratios were determined. As can be seen in Fig. 2A, the endogenous myocilin level in the lysate of human TM cells was increased by 2–3 fold upon treatment with proteasomal inhibitors, LCT and epoxomicin, and by 1.7–2.8 fold with lysosomal inhibitors. 3-MA did not alter the myocilin level. The level of myocilin present in the medium was similarly increased with proteasomal and lysosomal inhibitors but not with autophagic inhibitor 3-MA (Fig. 2B). Immunofluorescence experiments (Fig. 2C) confirmed the results presented in Fig. 2A. Compared to vehicle DMSO and 3-MA, the staining intensity or level of myocilin was higher following treatments of LCT, epoxomicin, NH_4_Cl and chroloquine (Fig. 2C). These data indicated that the endogenous myocilin was processed through both proteasomal and lysosomal pathways. Autophagy played a minimal, if any, role.

### Myocilin is ubiquitinated

**Figure 3:**
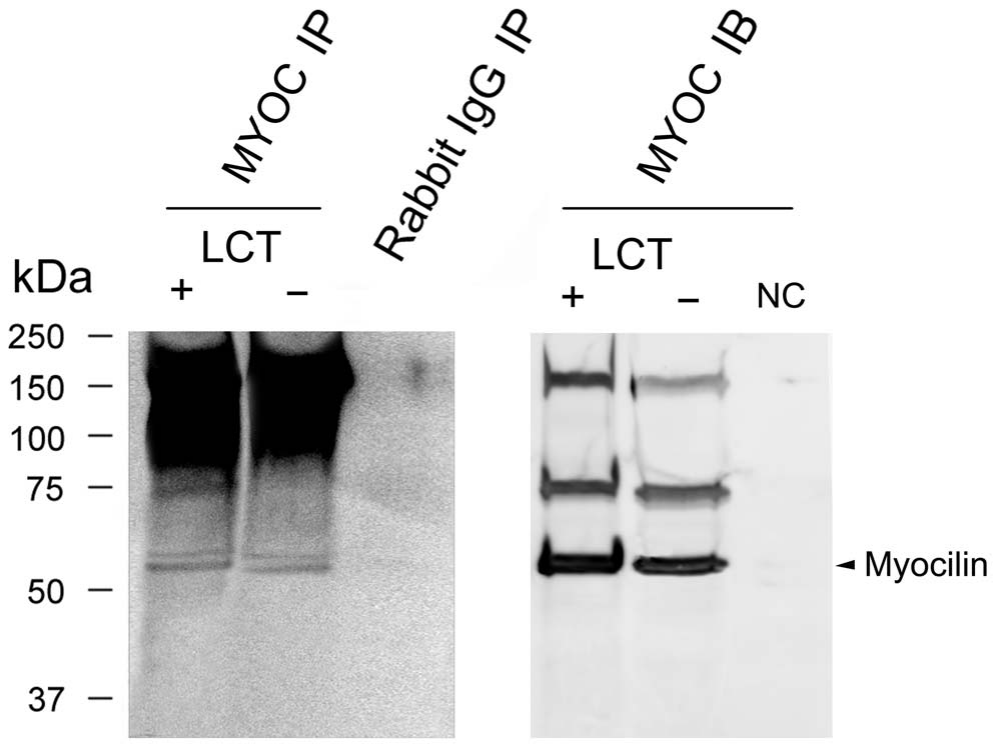
Myocilin is ubiquitinated. Total lysates from normal human TM cells without or with the LCT treatment were immunoprecipitated (IP) with rabbit anti-myocilin polyclonal antibody or normal rabbit IgG (as a negative control, NC) followed by immunoblotting (IB) with mouse anti-ubiquitin monoclonal antibody. Myocilin pull down by rabbit anti-myocilin (MYOC), but not the rabbit IgG control, showed multiple bands immunoreactive to anti-ubiquitin (left panel). The intensity of the ubiquitin-positive bands was enhanced by prior LCT treatment. The same blot was also probed with anti-myocilin (right panel) to verify the IP procedure. Arrow head, the 55/57-kDa myocilin bands.

To examine whether myocilin is ubiquitinated, lysates from human TM cells were immunoprecipitated with polyclonal anti-myocilin and immunoprobed with monoclonal anti-ubiquitin. Multiple bands immunoreactive to anti-ubiquitin (Fig. 3, left panel) were observed in the immunoprecipitated protein pool, indicating that the endogenous myocilin in human TM cells was ubiquitinated. Consistent with results shown in Fig. 2, the intensity of the ubiquitin-positive bands was enhanced by prior LCT treatment (Fig. 3, left panel). The same blot was also probed with anti-myocilin to verify the IP procedure (Fig. 3, right panel).

### Turnover of myocilin_WT_-GFP following photoactivation in human TM cells

**Figure 4:**
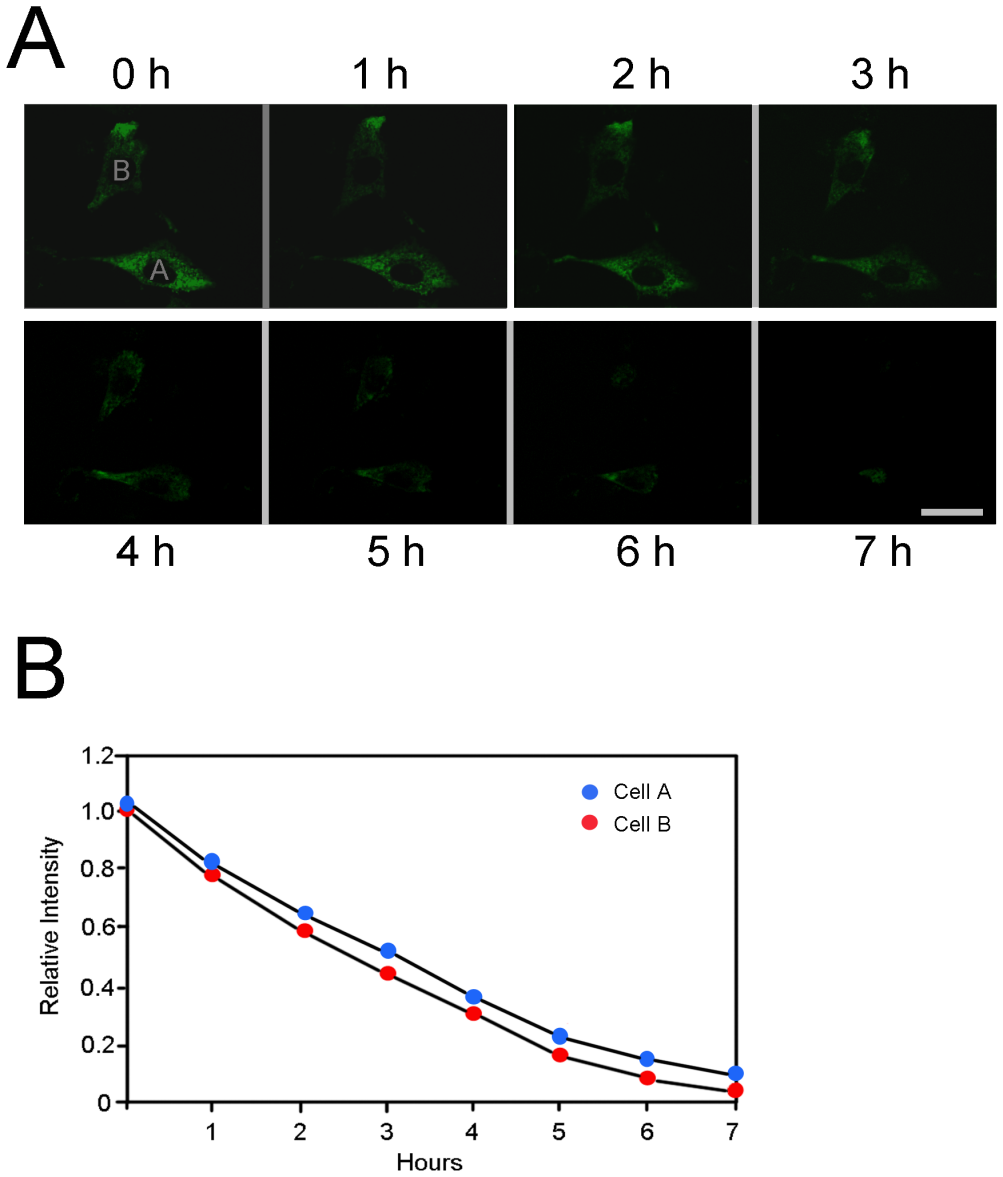
Photoactivation of myocilin_WT_-PAGFP. **A**. Human TM cells transfected with expression vector myocilin_WT_-PAGFP were imaged using 488-nm excitation. Selected cells were irradiated by 405-nm light. Green fluorescence, representing myocilin-GFP, was seen distributed in the entire cell upon irradiation. The field was monitored for 24 h and the fluorescence intensity at various time points was qualified by Image J. Images of two representative cells (cell A and cell B that displayed low intensity of green fluorescence) at time points 0, 1, 2, 3, 4, 5, 6, and 7 h are shown. **B**. The fluorescence intensity of myocilin_WT_-GFP in cells A and B was plotted against time to estimate the myocilin_WT_-GFP half-life.

Cells were transfected with myocilin_WT_-PAGFP (photoactivatable-green fluorescence protein). Immediately following photoactivation, green fluorescence, signaling the expression of myocilin_WT_-GFP, was seen filled in the entire cell. The intensity was high in some cells but moderate to low in others. The decrease or disappearance of the green fluorescence with time was monitored for 24 h with a Zeiss live cell imaging system (Fig. 4). It was found that the intensity of the green fluorescence sparked after irradiation was reduced with time. Overall, myocilin-GFP or the green fluorescence had an average of approximately 3 h half-life in the low-intensity or weakly fluorescent cells, similar to that of the endogenous myocilin found by Western blotting (Fig. 1A). The half-life in brightly and moderately fluorescent cells, representing overexpression situation, was longer (data not shown).

### Turnover of myocilin-GFP fusion proteins in inducible cells

**Figure 5:**
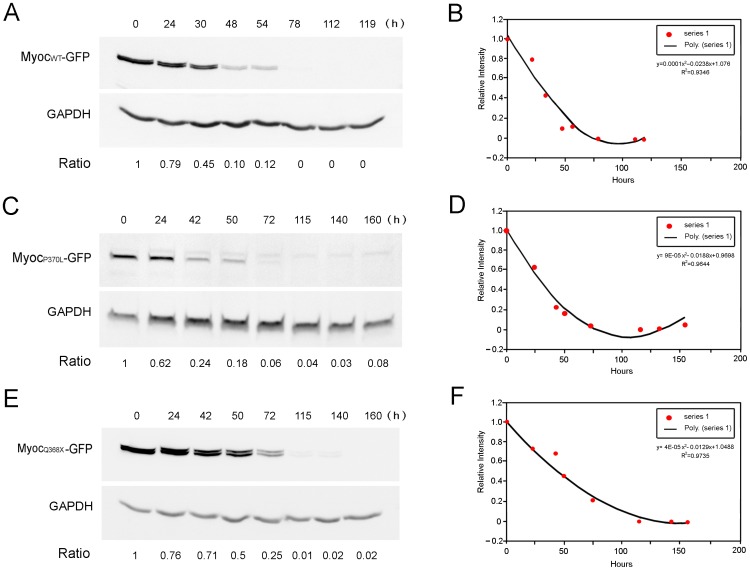
Turnover of overexpressed myocilin (wild-type or mutant)-GFP fusion proteins. Tet-on inducible RGC5 cells were treated with Dox (1 µg/ml) for 24 h to express a moderate level of wild-type (Myoc_WT_, **A**), mutant P370L (Myoc_P370L_, **C**) or Q368X (Myoc_Q368X_, **E**) myocilin-GFP fusion protein. After washing, cells were incubated in growth medium containing 1% fetal bovine serum (without Dox) and were harvested at various time points. Monesin (2 µM) was added to the wild-type myocilin-expressing cells to block the myocilin secretion. The levels of fusion proteins were measured by Western blotting. The relative intensity of the induced wild-type (**B**), P370L (**D**), and Q368X (**F**) myocilin-GFP to that of GAPDH was plotted against time to estimate the half-life. The half-life of wild-type-, P370L-, and Q368X-myocilin-GFP was approximately 24, 42, and 50 h, respectively. The turnover rate of overexpressed wild-type myocilin and mutants was much slower than that of the endogenous myocilin, suggesting that the protein processing was altered upon myocilin overexpression or mutation. All experiments were repeated at least 3 times, yielding similar results.

Tet-on inducible RGC5 cells were treated with Dox (1 µg/ml) for 24 h to express moderate levels of wild-type, mutant P370L or Q368X myocilin-GFP fusion proteins. The half-life of overexpressed wild-type-, P370L-, and Q368X-myocilin-GFP in induced cells was estimated to be 24, 42, and 50 h (Fig. 5), respectively, much lengthened compared to that of the endogenous myocilin (Fig. 1B), suggesting that the protein processing was altered upon myocilin overexpression or mutation.

### Effects of inhibitors on levels of overexpressed myocilin-GFP fusion proteins

**Figure 6:**
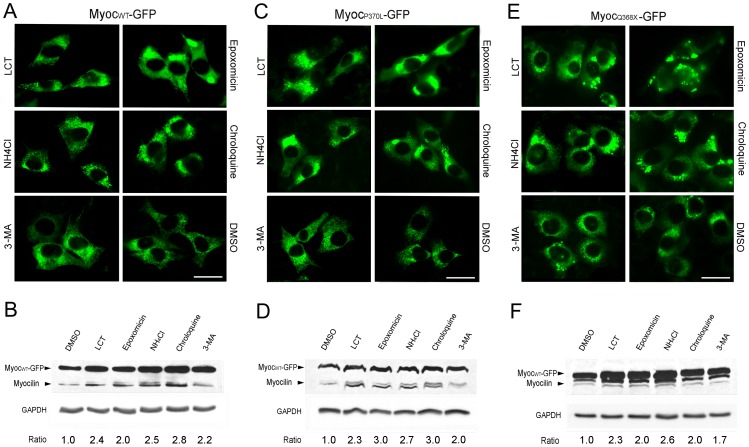
Effects of inhibitors on levels of expressed myocilin-GFP in inducible cells. Fluorescence images of myocilin_WT_-GFP (Myoc_WT_-GFP, **A**), myocilin_P370L_-GFP (Myoc_P370L_-GFP, **C**), myocilin_Q368X_-GFP (Myoc_Q368X_-GFP, **E**)-expressing-RGC5 cells treated with various inhibitors are shown. Scale bar, 20 µm. For Western blotting, RGC5 cells induced by Dox for 24 h to express myocilin_WT_-GFP (**B**), myocilin_P370L_-GFP (**D**), and myocilin_Q368X_-GFP (**F**) were treated for 16 h with vehicle DMSO, or proteasomal (LCT and epoxomicin), lysosomal (NH_4_Cl and chloroquin) or autophagic (3-MA) inhibitors. Proteins (25 µg) in cell lysates were immunoblotted with anti-myocilin or anti-GAPDH. Protein bands for the endogenous myocilin (55/57 kDa) and myocilin-GFP (∼100 kDa) were seen. Densitometry was performed. The myocilin-GFP/GAPDH relative to the DMSO control ratios are presented. All experiments were repeated at least 3 times.

Tet-on inducible stable RGC5 cells, after induction with Dox for 24 h were treated for 16 h with DMSO, H_2_O, LCT, epoxomicin, NH_4_Cl, chloroquin, or 3-MA. The fluorescence intensity of the induced wild-type and mutant myocilin-GFP fusion proteins was seen increased not only by proteasomal and lysosomal inhibitors but also by autophagic inhibitor 3-MA (Fig. 6A, C, E). Western blotting quantified that the increase of myocilin-GFP level following treatment of proteasomal and lysosomal inhibitors was by 2-3 fold and that by 3-MA was 1.7-2.2 fold (Fig. 6B, D, F). These results suggested that in addition to ubiquitin-proteasome and lysosomal pathways, autophagy was also involved in the processing of wild-type and mutant myocilin-GFP in inducible cells.

### Reduced proteasome activity in myocilin wild-type and mutant overexpressing cells

**Figure 7:**
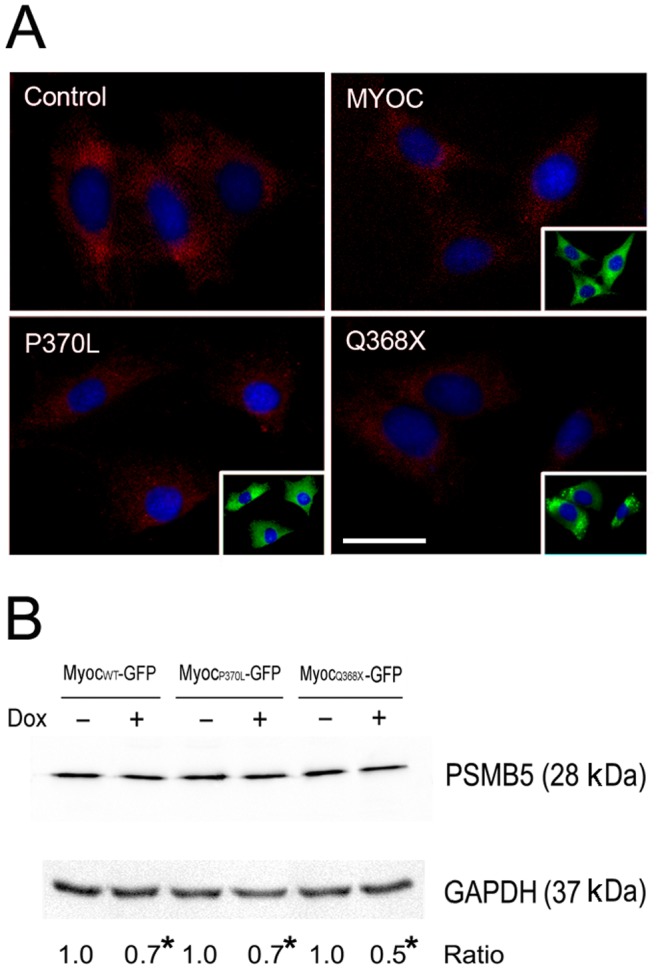
PSMB5 levels in inducible cells. **A**. PSMB5 immunostaining (in red) in inducible, stable RGC5 cells. The cells were induced by Dox for 24 h to express moderate levels of myocilin_WT_-GFP (MYOC), myocilin_P370L_-GFP (P370L) or myocilin_Q368X_-GFP (Q368X). The fusion protein-expressing, green fluorescent cells are shown in insets. There was no green fluorescence, as expected, in non-induced control cells. Note a reduced PSMB5 staining intensity in myocilin-GFP-expressing green cells compared with non-induced cells. Scale bar, 10 µm. **B**. Western blotting for PSMB5 protein level. RGC5 cells were induced for 24 h to express myocilin_WT_ (Myoc_WT_)-, myocilin_P370L_ (Myoc_P370L_)-, and myocilin_Q368X_ (Myoc_Q368X_)-GFP. Total cell lysate was subject to SDS-PAGE and immunoblotting using anti-PSMB5 or anti-GAPDH. The PSMB5/GAPDH ratios in induced cells relative those of non-induced controls are presented. *, P<0.002 compared to non-induced controls. All experiments were repeated at least 3 times, yielding similar results.

Tet-on inducible RGC5 cell lines treated with Dox for 24 h to induce moderate level expression of wild-type, P370L and Q368X myocilin-GFP were immunostained for PSMB5 as an indication of proteasome activity [45]. The staining intensity in green myocilin-overexpressing RGC5 cells was reduced compared to non-induced cells (Fig. 7A). Western blot analyses (Fig. 7B) indicated that the PSMB5 protein level was decreased by 30–50% (the values for wild-type, P370L, and Q368X, relative to their respective non-induced controls were 0.71±0.05, 0.72±0.04, and 0.50±0.07 respectively, n = 3, P<0.002).

### Induction of autophagy in myocilin overexpressing cells

**Figure 8:**
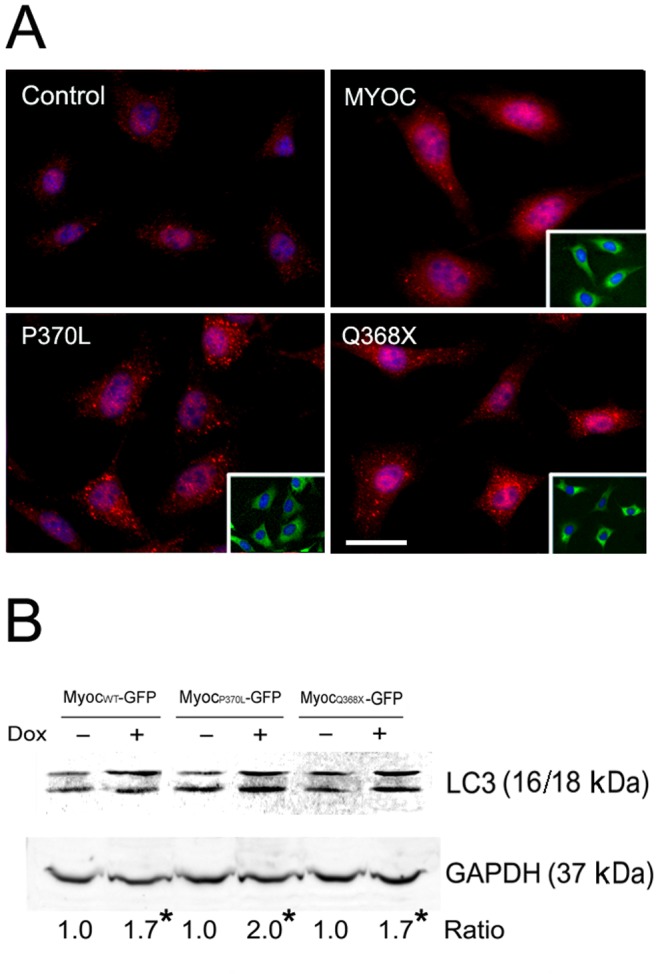
LC3 levels in inducible cells. **A**. LC3 immunostaining (in red) in inducible, stable RGC5 cells. The cells were induced by Dox for 24 h to express myocilin_WT_-GFP (MYOC), myocilin_P370L_-GFP (P370L), or myocilin_Q368X_-GFP (Q368X). The fusion protein-expressing, green fluorescent cells are shown in insets. There was no green fluorescence, as expected, in non-induced (control) cells. Note an increased LC3 staining intensity in myocilin-GFP-expressing green cells compared with non-induced cells. Scale bar, 10 µm. **B**. Western blotting for LC3 protein level. RGC5 cells were induced for 24 h to express myocilin_WT_ (Myoc_WT_)-, myocilin_P370L_ (Myoc_P370L_)-, or myocilin_Q368X_ (Myoc_Q368X_)-GFP. Total cell lysate was subject to SDS-PAGE and immunoblotting using anti-LC3 or anti-GAPDH. Both LC3-I and LC3-II protein bands were detected. The LC3/GAPDH ratios in induced cells relative those of non-induced controls are presented. *, P<0.0046 compared to non-induced controls. All experiments were repeated at least 3 times, yielding similar results.

Following myocilin-GFP induction, inducible RGC5 cells were stained for autophagic marker, LC3. The intensity of LC3 staining in myocilin-expressing green cells was found stronger than that seen in non-induced controls (Fig. 8A).

LC3 exists in two forms. LC3-I (18 kDa) is cytosolic and LC3-II (16 kDa) is lipidated (conjugated to phosphatidylethanolamine) that inserts into the membrane. The amount of LC3-II is correlated with the extent of autophagosome formation and increasing levels of LC3-II on immunoblots have been used to document induction of autophagy [43]. In RGC5 cells, the level of LC3 protein was found increased by 70 to 100% (the values for wild-type, P370L, and Q368X, relative to their respective non-induced controls were 1.69±0.07, 2.00±0.01, and 1.71±0.07 respectively, n = 3, P<0.0046) by Western blotting upon overexpression of wild-type and mutant myocilin (Fig. 8B).

## Discussion

Proper turnover of cellular proteins is of vital importance. Among the mechanisms for protein processing, the proteasome-ubiquitin pathway predominantly degrade short-lived nuclear and cytosolic proteins [31], [32], [34], [35]. Protein degradation via ubiquitin-proteasome pathway is a temporally controlled and tightly regulated process that involves covalent linking of a single or multiple molecules of ubiquitin to a target protein. The ubiquitinated protein is then marked for degradation by the multi-subunit 26S proteasome complex. The autophagy-independent lysosomal pathway is another pathway known to degrade extracellular proteins such as low density lipoprotein core particle [46] as well as endocytosed material such as GTPase RhoB [47] and membrane proteins [32], [33]. In addition, the bulk degradation of long-lived cytoplasmic proteins or organelles is mediated largely by autophagy and the autophagy-dependent lysosomes [36], [37].

The present study demonstrates that myocilin is a short-lived protein. Its half-life is determined to be approximately 3 h by turnover experiments (Figs. 1A and 1B), using lysates from a pool of human TM cells or RGC5 cells. By photoactivation experiment (Fig. 4), in which myocilin-GFP molecules, “turned-on” by photoactivation within a single living cell [38], [48], were monitored with live cell imaging, also estimated that the half-life of intracellular myocilin is around 3 h when the myocilin expression was low or seemingly at the endogenous level.

A new finding is that the endogenous myocilin is processed through both proteasomal and lysosomal pathways in human TM cells. Autophagy had a rather minor role. This notion stems from the finding that proteasomal and lysosomal inhibition led to an increase in the endogenous myocilin level, while autophagic inhibition had little effects (Fig. 2). The endogenous myocilin was found ubiquitinated (Fig. 3), further supporting the ubiquitin-proteasome involvement. In addition, the involvement of ubiquitin-proteasome pathway is consistent with our result of a short half-life of the endogenous myocilin (Figs. 1A, 1B, and 4).

The involvement of lysosomal pathway in the myocilin processing is unexpected. On the other hand, myocilin, while dynamic with a short half-life, is a membrane protein [16] associated with ER [49]. It has been shown that proteins to be degraded may be packed in ER membrane and the resulting vesicles are directly diverted into lysosomes [50]. Misfolded or inappropriately expressed proteins accumulated in the ER are also likely to be degraded in the lysosome compartment [51]. In the literature, proteins including gap junction proteins connexins [42], [52], epidermal growth factor [53], and factor VIII [54] have been shown to utilize both proteasomal and lysosomal systems for their processing and degradation. Myocilin can now be added to the list. Cooperation between lysosomes and proteasomes has also been seen for rapid removal of moderately or heavily oxidized proteins [55].

Our study further indicates that upon myocilin overexpression or mutation in inducible RGC5 cells, the turnover rate was much prolonged (Fig. 5). RGC5, an immortalized cell line established by transforming postnatal day 1 rat retinal cells with E1A adenovirus [56], [57], has been used widely and extensively as a model of RGCs for various investigations [39], [58]. However, a re-characterization by Van Bergen et al. [59] led to the conclusion that this cell line was of mouse origin rather than rat. More recently, an investigation by Krishnamoorthy et al. [39] indicated that the RGC5 cell line shared approximately 95% genetic markers with a mouse derived photoreceptor 661W cell line. Used not as a surrogate of RGCs, but as a vehicle, Tet-on inducible stable RGC5 cells were generated to facilitate studies of myocilin and its mutants in the present investigation. The wild-type and mutant myocilin proteins overexpressed upon Dox induction in inducible RGC5 cell lines appeared to be processed through not only proteasomal and lysosomal pathways but also by autophagy (Fig. 6). In accordance with this finding, the level of LC3, autophagic marker, was found induced as evidenced by an increased immunostaining and a higher LC3 level (Fig. 8). The proteasome activity was decreased (Fig. 7), perhaps because the system was overwhelmed and became dysfunctional by the overexpressed proteins. The suppression of proteasomal activity could further lead to the accumulation of the potentially cytotoxic proteins in the cells [60], [61]. In response, pathways such as autophagy, which serves as a default mechanism, may be activated to compensate for the reduced proteasome-mediated degradation of accumulated proteins. In the literature, cross-talk between the ubiquitin-proteasome system and the autophagy pathway has been reported [62], [63]. Activation of autophagy by proteasomal inhibition has occurred in many instances [64], [65].

An altered function of the ubiquitin-proteasome system has been linked to neurodegenerative diseases including Alzheimer's [66] and Parkinson's [67] diseases. A compromised ubiquitin-proteasome system is also seen upon myocilin overexpression or mutation. The similarity suggests that myocilin-related glaucoma shares common features with neurodegenerative diseases. Notably, upregulation or mutation of optineurin, product of another glaucoma gene, also leads to inhibition of the proteasomal activity and induction of autophagy [68]. Furthermore, overexpression of wild-type myocilin in the Drosophila eye has been shown to cause aggregation of myocilin [69] and activation of UPR [70]. It was postulated that the impaired ability of the UPR to eliminate damage or proteins misfolding such as myocilin would induce ER stress, resulting in functional deficiency of TM cells and pathologic consequences [70].

Taken together, the current study provides evidence that in normal homeostatic situation, the turnover of endogenous myocilin involves both proteasomal and autophagy-independent lysosomal pathways. When myocilin is upregulated or mutated, the proteasome function is compromised and autophagy is activated. Knowledge of the degradation pathways acting on myocilin can help in design of novel therapeutic strategies for myocilin-related glaucoma. For example, proteasome activity can be promoted by overexpression of proteasome subunit or molecular chaperones.

## Materials and Methods

### Cell cultures

Human TM cells were derived from Eyebank eyes of 20, 22-, and 35-year-old donors. RGC5 cells were obtained from the departmental core facility at the University of Illinois at Chicago, deposited by Dr. Paul Knepper [71] and generously provided originally by Dr. Neeraj Agarwal [56], [57]. Human TM and RGC5 cells were grown in Dulbecco's modified Eagle's medium (DMEM) supplemented with 10% fetal bovine serum (FBS) and antibiotics. Tet-on wild-type myocilin (MYOC_WT_, 1–504 amino acids)-GFP inducible stable RGC5 cell line was established as previously described [40]. Briefly, a plasmid vector pTRE-MYOC-EGFP-INS-rtTA-IRES-hyg-pcDNA3.1z which contains both tetracycline regulatory and responsive components based on the Clontech's Tet-on advance system (Clontech, Mountain View, CA) was constructed. Two other plasmid vectors containing P370L and Q368X mutations were similarly prepared. RGC5 cells were transfected with these constructs and selected in hygromycin (100 µg/ml)-containing medium. The selected cells were maintained in Dulbecco's Modified Eagle Medium (DMEM) complete medium with 10% Tet system certified fetal bovine serum (Clontech), essential and nonessential amino acids, and antibiotics. To induce expression of MYOC_WT_-GFP, MYOC_P370L_-GFP, and MYOC_Q368X_-GFP, cells were treated for 48 h with Dox (1 µg/ml) (Clontech) in DMEM complete medium. Cells were screened for high, moderate, or low fusion gene expression after induction by fluorescence microscopy.

### Turnover of myocilin

To examine the turnover of the endogenous myocilin and β-catenin, human TM cells or RGC5 cells in 6-well plates (300,000 cells/well) were incubated in serum-free growth medium containing CHX (5 µg/ml, to inhibit protein synthesis) and monesin (2 µm, to block protein secretion) and harvested every hour thereafter. The cells were lysed with lysis buffer (250 mM NaCl, 50 mM Tris/HCl, pH 7.5, 5 mM EDTA, 0.5% Nonidet P40) supplemented with protease inhibitor cocktail (Sigma, St. Louis, MO). Protein concentration was determined by bicinchoninic acid protein assay (Pierce, Rockford, IL). Total cell lysate was then subjected to sodium dodecyl sulfate-polyacrylamide gel electrophoresis (SDS-PAGE) under reducing conditions. The proteins were transferred to nitrocellulose membrane and the level of endogenous myocilin was assessed by Western blotting using monoclonal anti-myocilin antibody 1.1 [44] (a generous gift from Dr. Michael Fautsch, Mayo Clinic). The membrane was also immunoblotted with polyclonal anti-GAPDH (1∶5000, Trevigen, Gaithersburg, MD) for loading control. For turnover of β-catenin, rabbit anti-β-catenin polyclonal antibody (Santa Cruz Biotechnology, Dallas, Texas) was used. Immunoreactive protein bands were detected by chemiluminescence using SuperSignal Substrate (Pierce). Densitometry was performed. The band intensity of the endogenous myocilin and β-catenin was normalized to that of GAPDH. The myocilin/GAPDH or β-catenin/GAPDH ratios determined were plotted against time points. The polynomial equation that fitted the curve was used to estimate the half-life of the protein.

To investigate the turnover rate of overexpressed myocilin wild-type or mutant (P370L or Q368X)-GFP proteins, Tet-on inducible myocilin-GFP (wild-type, P370L or Q368X)-expressing RGC5 stable cells were plated in 6-well plates and induced 24 h with Dox (1 µg/ml). Cells were washed with phosphate buffered saline three times and incubated in growth medium containing 1% of fetal bovine serum (without Dox). To wild-type myocilin-expressing cells, monesin (2 µM) was also added to block the myocilin secretion. Cells were harvested at various time points and subjected to SDS-PAGE and immunoblotting with anti-myocilin and anti-GAPDH. The level of myocilin-GFP fusion proteins was normalized to that of GAPDH. The ratios were plotted against time points and polynomial equations that fitted the curves were used to estimate the half-lives of myocilin fusion proteins. All experiments were repeated at least three times.

### Effects of inhibitors on myocilin levels

To determine the effects of various inhibitors on levels of the endogenous myocilin, human TM cells were treated for 16 h with vehicle DMSO or H_2_O, proteasomal inhibitors lactacystin (LCT, 1 µM) and epoxomicin (5 µM), lysosomal inhibitors NH_4_Cl (1 mM), and chloroquin (10 µM), or autophagic inhibitor 3-MA (5 mM). Total cell lysate (25 µg) was collected for immunoblotting with anti-myocilin antibody 1.1 and anti-GAPDH. The level of the endogenous myocilin was normalized to that of GAPDH.

The level of induced wild-type fusion protein as affected by the inhibitors in inducible RGC5 cells was also assessed by Western blotting. Total cell lysates were dissolved in 10% SDS-PAGE and immunoblotted with monoclonal anti-myocilin 1.1 and anti-GAPDH as above.

All experiments were repeated at least three times.

### Immunofluorescence for myocilin

Human TM cells or induced RGC5 cells treated with proteasomal, autophagic, or lysosomal inhibitors as above were fixed and stained with monoclonal anti-myocilin. FITC-goat anti-mouse IgG (Jackson ImmunoResearch, West Grove, PA; 1∶200) was used as the secondary antibody. The slides were mounted in Vectashield (Vector Laboratories, Burlingame, CA) with 4',6-diamidino-2-phenylindole (DAPI). Photography was carried out using a 63× oil objective on an Axioscope (Carl Zeiss MicroImaging, Thornwood, NY) with the aid of Metamorph software (Molecular Devices, Downingtown, PA).

### PSMB5 and LC3 levels

Tet-on inducible RGC5 cell lines were treated with Dox for 24 h to induce moderate expression of wild-type, P370L, or Q368X myocilin-GFP. The total cell lysate was subjected to Western blotting using rabbit anti-PSMB5 (Abcam, Cambridge, MA), rabbit anti-LC3 (MBL, Farmingdale, NY), or rabbit anti-GAPDH. The level of PSMB5 or LC3 normalized to that of GAPDH in induced cells was compared to that in non-induced control cells.

For immunofluorescence, cells induced with Dox were fixed and stained with rabbit anti-PSMB5 or rabbit anti-LC3. Cy5-goat anti-rabbit IgG (Jackson ImmunoResearch, 1∶200) was used as the secondary antibody.

### Immunoprecipitation

Lysates from human TM cells untreated or treated with 1 µM LCT for 16 h were immunoblotted using polyclonal anti-myocilin or monoclonal anti-ubiquitin (1∶2000, Biomol, Enzo Life Sciences). Lysates were also immunoprecipitated with polyclonal anti-N-terminal-myocilin (a generous gift from Dr. W. Daniel Stamer, Duke University) or rabbit normal IgG (negative control) using the Catch and Release kit (Millipore, Billerica, MA). The proteins pulled down were subjected to SDS-PAGE under reducing conditions. The ubiquitinated proteins were detected with mouse anti-ubiquitin antibody.

### Photoactivation and live cell imaging

Construct myocilin_WT_ tagged with PAGFP (Addgene, Cambridge, MA) was made by insertion of NheI+BamHI digested myocilin_WT_ gene (full length, 1–504 amino acids) into NheI+BamHI linearized pPAGFP-N1 vector (Addgene, Cambridge, MA) and transfected into human TM cells. The transfected cells were observed on Zeiss Cell Observer SD (spinning disk) system 24 h post transfection. Myocilin_WT_-PAGFP was irradiated by 405-nm light and excited by 488-nm light. Live-cell videos were recorded up to 24 h. Fluorescence of the excited cells at different time points was analyzed by Image J software to quantify the turnover time of myocilin-PAGFP fusion protein. At least 20 cells that displayed low-intensity green fluorescence were analyzed. The relative intensities of fluorescence were plotted with the time and the half-life of myocilin was estimated.

### Statistical analysis

All experiments were carried out at least 3 times. One-way ANOVA was performed as a statistical measure for significance of the data. Statistical significance was noted if P<0.05.
